# Salute to the sun: a new dawn in yoga therapy for breast cancer

**DOI:** 10.1002/jmrs.218

**Published:** 2017-01-30

**Authors:** Melissa Galliford, Stephanie Robinson, Pete Bridge, MaryAnn Carmichael

**Affiliations:** ^1^ Genesis Cancer Care Bunbury Western Australia Australia; ^2^ Radiation Oncology Centres Brisbane Queensland Australia; ^3^ University of Liverpool Liverpool United Kingdom; ^4^ Queensland University of Technology Brisbane Queensland Australia

**Keywords:** Breast, cancer, quality of life, radiation therapy, yoga

## Abstract

**Introduction:**

Interest in the application of yoga for health benefits in western medicine is growing rapidly, with a significant rise in publications. The purpose of this systematic review is to determine whether the inclusion of yoga therapy to the treatment of breast cancer can improve the patient's physical and psychosocial quality of life (QoL).

**Methods:**

A search of peer reviewed journal articles published between January 2009 and July 2014 was conducted. Studies were included if they had more than 15 study participants, included interventions such as mindfulness‐based stress reduction (MBSR) or yoga therapy with or without comparison groups and had stated physical or psychological outcomes.

**Results:**

Screening identified 38 appropriate articles. The most reported psychosocial benefits of yoga therapy were anxiety, emotional and social functioning, stress, depression and global QoL. The most reported physical benefits of yoga therapy were improved salivary cortisol readings, sleep quality and lymphocyte apoptosis. Benefits in these areas were linked strongly with the yoga interventions, in addition to significant improvement in overall QoL.

**Conclusion:**

The evidence supports the use of yoga therapy to improve the physical and psychosocial QoL for breast cancer patients with a range of benefits relevant to radiation therapy. Future studies are recommended to confirm these benefits. Evidence‐based recommendations for implementation of a yoga therapy programme have been derived and included within this review. Long‐term follow‐up is necessary with these programmes to assess the efficacy of the yoga intervention in terms of sustainability and patient outcomes.

## Introduction

Cancer incidence projections for Australia between 2011 and 2020 predict that 17 210 cases of breast cancer will be diagnosed in 2020.^1^ Radiation therapy can offer promising survival rates for women with this disease but can result in a range of burdening physical and psychological side effects.^2^, ^3^, ^4^, ^5^ It is important that efforts to improve patient quality of life (QoL) and the overall survivorship pathway keep pace with technical developments; yoga potentially has a role to play here.

Yoga traces its roots back to India from 5000 B.C.E.^6^ The practice as it is taught today encourages health and relaxation through a combination of mind‐body techniques, including specific postures, breathing techniques, meditation, chants, and wisdom teachings.^6^ Interest in the physical and psychosocial health benefits of yoga in western medicine is growing rapidly, at the rate of 24 published studies on PubMed per year since 2007.^6^ The purpose of this systematic review is to determine whether complementary yoga therapy can improve breast cancer patients' QoL from both physical and psychosocial perspectives and derive best practice guidelines and recommendations.

## Methods

**Figure 1:**
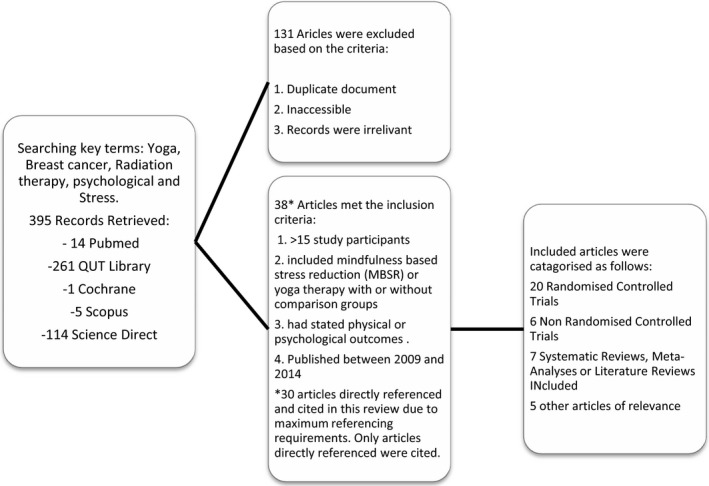
Flow chart highlighting results of the literature search.

A comprehensive search strategy was performed using Scopus, Medline, PubMed, ScienceDirect, Cochrane and ProQuest to ensure relevance to health research. Publications were selected from peer reviewed journals published after January 2009 and included if they contained 15 or more participants. See Figure 1 for search terms and inclusion criteria.

The authors utilised a blinded and duplicated 2‐stage screening approach to select and categorise appropriate evidence. Initially abstract review was performed to ensure the inclusion criteria were met and to exclude published ‘abstracts only’, letters and comments, case reports and animal studies. Following this, a full‐text article screening was performed to ensure relevance of content. For inclusion, articles must have reported outcomes related to physical or psychosocial aspects of QoL for breast cancer patients. Interventions included Mindfulness‐Based Stress Reduction (MBSR) or yoga therapy, with or without comparison to another intervention (i.e.: wait list, control group, usual care). There were no additional limitations on study design. Critical review and scoring of the resulting selected articles was performed independently by two researchers to reduce observer bias. A structured approach to critical review was utilised based on the SIGN critical appraisal checklists.^7^

## Results

### Characteristics of included studies

The characteristics of the studies are summarised in Table 1. The number of participants in randomised controlled trials (RCTs) ranged between 18 and 410. Based on the average ages presented in 12 of the RCTs, the mean age of participants was 58.3 years. Of the five non‐randomised trials (NRTs) included in this study, the number of participants ranged between 15 and 286. Based on the average ages presented in the five NRTs, the mean age of participants was 50.3 years. All participants in the RCTs and NRTs who were breast cancer patients were documented to be women, with the exception of the study by Park et al.^8^ in which 20% (*n* = 9) of the participants were men.

**Table 1:** Summary of yoga interventions in included studies

Aspect	Summary
Timing	Yoga introduced two – 18 months post treatment. *n* = 7 Yoga introduced in concurrence with radiation therapy. *n* = 6 Yoga introduced before radiation therapy. *n* = 2
Frequency	Median yoga intervention = 6 weeks Range of yoga intervention = 4 weeks–6 months At home practice was encouraged. *n* = 10
Yoga practices	Combination of breath, movement and meditation. *n* = 10 Movement and meditation only. *n* = 3 Breath and meditation only. *n* = 1 Meditation only. *n* = 1
Comparison groups^a^	Group therapy. *n* = 5 Control groups. *n* = 3 Usual care and wait list groups. *n* = 6 Nutritional care group. *n* = 1 Stress management seminar group. *n* = 1 Stretch group. *n* = 1 No comparison group. *n* = 1

*n*, total number of studies.

a Some studies included more than one comparison group type.

### Yoga interventions

Please refer to Table 1 for a summary of yoga intervention aspects across included studies.

### Outcome measures

Four physical and 11 psychosocial outcomes were noted in the RCTs accepted for this review. The physical outcomes included salivary diurnal cortisol, stress, sleep quality and fatigue, as well as hot flush frequency and severity. Psychosocial outcomes included anxiety changes, emotional and social functioning, overall QoL, stress levels and depression. Additional psychosocial outcomes such as fear of cancer recurrence, hostility, mood disturbances, paranoid ideations, vitality and increased meaning to life were examined by one or two studies.

## Discussion

### Stress

Carlson utilised the C‐SOSI Questionnaire to assess the stress levels experienced by their patients and noted significant improvement for the intervention group compared to the control (*P* = 0.009).^9^ Additional studies suggest that yoga therapy is up to twice as beneficial at reducing stress and distress for breast cancers.^10^, ^11^, ^12^, ^13^ Effective stress reduction can help patients to identify and evaluate problems with a greater emotional equilibrium and enable active pursuit of personal health objectives.^14^ The benefits of reduced stress will improve the experience of radiation therapy for breast cancer patients as well as promote recovery towards the end of and completion of treatment.

Diurnal salivary cortisol levels are directly associated with chronic stress.^15^ The earliest published literature included in this review demonstrated a significant decrease in mean salivary cortisol levels at 6 a.m. (*t* = 4.21, *P* < 0.001) compared to the control group (*P* = 0.009), and pooled diurnal mean cortisol (*t* = 2.79, *P* = 0.01) for the yoga intervention group using a paired‐samples *t*‐test.^16^ This study was, however, limited due to the inequality in contact duration of interventions. Banasik et al.^15^ supported these findings with statistically significant improvement in morning and evening salivary cortisol concentrations (*P* = 0.018 and 0.028 respectively). These results were, however, limited by the small sample size of the study; collection of saliva samples over several consecutive days would have improved accuracy.

Two recently published studies both collected saliva samples over multiple days, increasing the reliability of their findings. Chandwani et al.^17^ reported that the yoga group had a significantly steeper slope, indicating reduced stress, compared to both the active stretching and the waitlist control groups. Bower et al.^18^ reported that, based on the diurnal salivary samples obtained after their 12 weeks yoga intervention, there was no significant difference between yoga and control groups post intervention compared to baseline. However, it was suggested that blood cortisol levels may be more helpful in assessing the impact of yoga. The results of this study indicate that yoga may influence the sensitivity of anti‐inflammatory glucocorticoid receptor (GR), rather than daily cortisol production. Their study also confirmed that the yoga therapy intervention increased GR activity and that further research could determine the impact yoga may have on this specific aspect of the HPA axis.^18^

Of the four RCTs that performed diurnal salivary cortisol as an outcome measure, three required eligible participants to be post treatment. This may have been an influencing factor in the differences between study findings as it does appear that the yoga intervention during treatment causes more significant improvement in the steepness of cortisol slopes, or decreases in cortisol levels.^16^, ^17^ More consistent reporting methods of salivary cortisol levels with ng/dL per hour should be used in future studies.

### Overall quality of life

All studies used the EORTC or FACT questionnaires to assess the patient's QoL, with the exception of Vadiraja who combined a number of factor scores to determine the overall QoL.^16^ One study discovered a significantly strong correlation between increased QoL scores and frequency of yoga intervention (*P* = <0.05).^19^ Collaboratively, these results suggest that yoga is very effective in improving the QoL of breast cancer patients. These conclusions are further supported by additional studies.^10^, ^11^, ^12^ One review of 12 RCTs deduced that yoga caused short‐term improvements in global health‐related QoL (*P* = 0.04), social (*P* < 0.01), and spiritual well‐being (*P* = 0.01); however, subgroup analysis revealed evidence of efficacy only during active cancer treatment.^11^

### Sleep quality

Although all studies that measured fatigue pre‐ and post intervention recorded significant improvement, cohorts from studies that received yoga interventions throughout treatment achieved more significantly improved scores on the Pittsburg Sleep Quality Tests. Further study is required to determine if this is dependent on the comparatively lower baseline scores. Danhauer et al.^20^ was prompt to acknowledge that future research should provide stronger evidence to determine when a yoga therapy intervention would be the most useful for breast cancer patients.

### Lymphocyte apoptosis

A NRT cross‐sectional pilot study revealed that percentage apoptosis and qualitative DNA damage were lowest among senior yoga practitioners, and highest among healthy non‐yoga volunteers, with the percentage of partially damaged DNA (‘comet’) occurrences highest among breast cancer patients.^21^ It was acknowledged that this high percentage of comet occurrences in breast cancer patients could be a direct result of treatment‐related DNA damage; in theory regular yoga practice could address this.^21^ It could, therefore, be pertinent for breast cancer survivors to start yoga therapy in the early stages of their cancer trajectory, and also continue well past the conclusion of active treatment. The associated stress reduction and resulting metabolic and oxidative homeostasis should improve essential restorative cellular processes.^21^ Yoga therapy could therefore reduce the effects of age‐dependant DNA damage and repair capacity, and assist in maintaining the QoL for cancer survivors.

### Anxiety

Evidence has shown that the addition of MBSR or yoga therapy to breast cancer treatment reduced incidence and severity of anxiety. Three of five studies investigating anxiety showed improvements in levels of general anxiety among yoga therapy cohorts.^4^, ^9^, ^10^, ^14^, ^16^ The remaining two large‐scale studies divided the term anxiety into state and trait anxiety.^4^, ^9^State anxiety refers to a temporary response to a threat, which subsides once the threat has been removed while trait anxiety refers to uncontrollable anxiety.^22^ Improvements in both states of anxiety were noted. These indications of reduced anxiety are supported by claims made by Buffart et al.,^10^ that large reductions in anxiety levels were associated with more frequent yoga therapy.

### Emotional and social functioning

Five studies found that patients engaged in the yoga programme scored higher in emotional and social functioning surveys and out‐performed comparison groups throughout the entire course of the intervention^3^, ^4^, ^5^, ^19^, ^30^ Two sets of results found that the comparison group, who were not exposed to yoga therapy, displayed decreased scores from baseline to post‐intervention, indicating that they experienced a negative effect on their social functioning levels.^16^, ^19^ The findings relating to emotional and social functioning are consistent with those stated within relevant meta‐analyses.^10^, ^11^, ^12^ The benefits of refined emotional and social functioning for breast cancer patients include improved coping strategies in addition to the ability to strengthen relationships with support groups. This is of particular importance for patients undergoing radiation therapy, typically at the end of their treatment journey. These patients are likely to be most fatigued and stressed so the need for adequate and close support services during this time is paramount.

### Depression

Reduced levels of depression and depressive symptoms were present in all studies investigating the psychosocial benefits of yoga therapy.^3^, ^4^, ^5^, ^8^, ^9^, ^10^, ^13^, ^14^, ^16^, ^20^, ^23^, ^25^, ^29^, ^30^ Chen et al.^23^ reported a strong correlation (*P* = 0.001) regarding the improvement of depression levels over time for both cohorts. Nevertheless, it is clear within their results that the yoga group was less depressed at both baseline and post‐intervention, suggesting that the addition of yoga therapy has the ability to accelerate the reduction of depression and depressive symptoms. Significant reductions in depression scores were also seen within the research of Buffart et al. and Cramer et al.^10^, ^11^ The concurrence of these results with the aforementioned meta‐analyses validates that depression levels among breast cancer patients can be improved with the inclusion of yoga therapy to conventional treatment.

### Yoga therapy implications

The literature supports the use of yoga therapy as a prophylactic intervention that can prevent persistent fatigue and preserve the long‐term QoL outcomes for patients.^12^, ^24^ Since pre‐treatment fatigue is a strong predictor of persistent fatigue, it would be sensible to implement yoga before treatment.^25^ Indeed, Slocum‐Gori et al.^26^ reported how patients were actively seeking guidance in this regard. It is clear that yoga therapy interventions should be offered at the beginning of the patients treatment and survivorship pathway. Additionally, it can be inferred from the increased relaxation achieved through yoga therapy that it should be implemented before or in concurrence with radiation therapy. The relaxed state of the patient may reduce the incidence of intrafractional motion and thereby improve treatment set up accuracy. One limiting factor to introducing the yoga programme adjacent to diagnosis is the potential for information overload, which can be associated with increased levels of stress.^26^ Concern about this issue was voiced by two of the subjects included in this study.

It is clear that yoga therapy should continue after treatment to ensure the benefits are fully experienced. The initial 12 months following completion of primary breast cancer treatment is the most difficult in which to begin efficient recovery.^25^ This is commonly due to unstable QoL experienced since diagnosis combined with physical side effects, depression and anxiety. The advantageous results that have been achieved with the addition of yoga therapy may promote recovery.

While yoga therapy will be beneficial to all breast cancer patients, extensive research suggests that the intervention will be most beneficial to those patients with existing low levels of optimism, QoL and interpersonal relationship issues.^27^ More frequent participation in yoga therapy is associated with greater benefits, therefore increased frequency of participation should be a primary focus among yoga therapy providers.^19^, ^27^

### Limitations of this report

The search criteria and focus have led to several limitations within this report. Limitation to English language publications may have omitted some potentially useful non‐English findings. Additionally, the conclusions are limited in validity to breast cancer patients only, although the findings do indicate further study in other disease sites.

Another limitation arises from methodological issues including lack of long‐term data and limited use of control groups. Few studies included in this review utilised control measures; these included health education or supportive therapy and rarely matched the frequency or duration of the yoga therapy. As such, results indicating the benefits of yoga may be partially due to the different intervention hours. Another common issue with complementary medicine is bias; inability to use blinding with subjects or participants limits achievable scores on quality criteria assessment.^11^, ^13^ Unfortunately, external assessment outcomes cannot adequately measure all of the benefits achieved; however, future studies could consider blinding of physical outcome assessment to provide some reduction in bias.^11^

Most of the underpinning evidence for the study derived data from mostly homogenous cohorts of well educated women with high socioeconomic status and therefore good access to health care. Although this cohort is representative of the wider demographic for breast cancer, with only one RCT explicitly including an ethnically diverse sample of participants, it is impossible to discount cultural influences from the findings.^11^ Evidence suggests that yoga appeals to women with more resources, specifically those with higher educational backgrounds and higher income.^28^ Thus, it may become important to develop pathways to making yoga more appealing to a broader range of patients.

Adherence to interventions is another common limitation correlating with poor results.^12^ This is compounded by study participants with worse initial QoL scores often experiencing lower adherence rates. Use of prophylactic yoga interventions simultaneously with active cancer treatments or targeting of support to low QoL patients may help to address this imbalance.

## Recommendations

Taking into account the diverse range of protocols utilised within the RCTs and NRTs included in this review and the suggestions within the literature, a recommended template protocol has been devised to allow yoga therapy interventions to be successful and beneficial for all involved. Table 2 summarises these findings.

**Table 2:** Recommendations for yoga therapy programme implementation

Aspect	Recommendation
Style	Suitable styles include Hatha, gentle restorative or yoga therapy
Timing	Yoga therapy should be introduced as soon as possible after diagnosis although introduction at any stage throughout the survivorship pathway will prove beneficial to most participants. Yoga therapy should parallel conventional treatment to increase coping mechanisms and vigour, and should continue until at least 6 months after adjuvant treatment. Ongoing inclusion of yoga into patients' lifestyles after this time frame is encouraged
Practice	Yoga programmes should comprise 3× 90‐min classes a week guided by a qualified yoga instructor. Home practice should be encouraged once the essential skills and safety of the practice have been established. Patients should have teaching aides available to encourage and guide home practice
Components	Programmes should focus on the three main components of yoga; postures, breathing and meditation. An important aspect of the classes should be meditation to improve mental health and coping strategies as well as introducing themes to guide patient recovery. Examples of themes are acceptance, listening to your body, promoting change, support and recovery.
Class Sequences	Poses and stretches should be kept similar each week with small, progressive increases in difficulty available to promote improvement. Each class should introduce a new mindfulness focus.
Modifications	Posture variations should accommodate patient limitations, with potential additional props including lavender pillows, blankets and bolsters for support. Use of an initial basic health assessment form should identify potential psychological or physical ailments which require attention during the programme
Instructors	Qualified instructors registered with National Yoga Teacher Training programmes should be utilised. All instructors should undertake annual professional development activities and CPR/First Aid refreshers.
Measurement	Use of a yoga log or diary to record frequency and duration of yoga therapy is recommended to monitor progress, effectiveness and record compliance. Participant feedback should be sought throughout the programme.

### Future study suggestions

It would be useful to determine the specific impact of yoga therapy on breast cancer patients undergoing radiation therapy. Both control (normal care) and yoga groups should be compared to quantify changes in patient relaxation during treatment set up, intrafractional motion, treatment‐related side effects and levels of anxiety. Due to the potential for increased accuracy of treatment, long‐term follow‐up data should be gathered to determine long‐term efficacy of the programme.^29^ This should include assessment of response to treatment, including recovery of treatment‐related side effects, and disease recurrences to identify any correlation with improved prognosis and survivorship pathway.

## Conclusion

The evidence within this review supports the use of yoga therapy to improve physical and psychosocial QoL for breast cancer patients undergoing radiation therapy. Adherence to the yoga therapy protocol included within this review is recommended. Future studies including long‐term follow‐up are recommended to assess the efficacy of the yoga intervention in terms of post‐treatment outcomes and sustainability.

## Conflict of interest

The authors declare no conflict of interest.
